# Machine learning in automatic detection of chordoma signature, postoperative residuals, and prognosis of skull base chordomas

**DOI:** 10.1038/s41698-026-01459-1

**Published:** 2026-05-22

**Authors:** Daniela Stastna, Richard Mannion, Robert Macfarlane, Patrick Axon, Neil Donnelly, James R. Tysome, Daniele Borsetto, Ryan Chrenek, Kaasinath Balagurunath, Wenya Linda Bi, Carleton E. Corrales, Ossama Al-Mefty, Timothy Smith, Ari Ercole, Jonathan Coles

**Affiliations:** 1 Skull Base Unit, Cambridge University Hospitals, Cambridge, UK; 2 Skull Base unit, Mass General Brigham Hospital, Harvard University, Boston, MA USA; 3 Perioperative, Acute, Critical Care and Emergency Medicine (PACE), Department of Medicine, Cambridge University Hospitals, Cambridge, UK

**Keywords:** Cancer, Medical research, Oncology

## Abstract

Skull base chordomas are rare, locally invasive tumors that remain a diagnostic and therapeutic challenge. We developed a machine-learning (ML) radiomics model to (i) distinguish chordoma from chondrosarcoma and skull base background, (ii) differentiate true postoperative residual tumor from treatment-related changes, and (iii) predict 2-year progression-free survival (PFS). In this retrospective, dual-center study, 61 patients underwent surgery between 1998 and 2023. Preoperative contrast-enhanced T1-weighted MRI images were pre-processed and segmented; data were augmented by 20%. ML models included nested cross-validated XGBoost and a 4-layer standard feedforward Multilayer Perceptron (MLP) (Python, Keras). The primary and secondary endpoints were diagnostic discrimination and residual-versus-treatment-related change classification; the exploratory endpoint was 2-year PFS prediction. XGBoost achieved diagnostic accuracy of 0.90 (95% CI: 0.84–0.96) in distinguishing chordoma from chondrosarcoma/skull base background, and residual-versus-change accuracy 0.91 (95% CI: 0.85–0.96). PFS prediction reached an accuracy of 0.87 (95% CI: 0.74–0.98). MLP showed comparable performance (diagnostic validation accuracy 0.89; residual classification 0.90; PFS 0.93). To our knowledge, this is the first dual-center MRI-based ML study to jointly address preoperative histologic discrimination, postoperative residual detection, and short-term PFS prediction in a small, heterogeneous cohort. These results support future clinical translation as a noninvasive decision-support tool for preoperative assessment, postoperative surveillance, and risk stratification.

## Introduction

Skull base chordomas are rare, locally destructive, and highly recurrent pathologies with an affinity for the clivus^1–16^. Chordomas originate from remnants of the notochord in the spheno-occipital synchondrosis. Their reported incidence is approximately 8 cases per 10 million people^1^. Skull base chordomas share a similar affinity for the clivus, radiological features, growth patterns, and clinical presentation with chondrosarcomas, making the distinction often challenging^3^. Moreover, chordomas and chondrosarcomas have similar demographic features, such as an incidence between the fourth and sixth decade, and equal gender distribution^2,3^. Chondrosarcomas typically arise from primitive mesenchymal cells and the cartilaginous matrix near the petro-occipital fissure or spheno-ethmoidal suture^2,3,11,12^.

Clival chordomas and chondrosarcomas exhibit locally invasive multidirectional growth. From a radiological perspective, both pathologies are osteolytic lesions that demonstrate predominantly high signal intensity on T2-weighted magnetic resonance imaging (MRI) sequences, intermediate-to-low signal intensity on T1-weighted MRI sequences (T1wMRI), and heterogeneous contrast-enhancement. Diffusion-weighted MRI has shown some capacity for distinguishing between classic chordoma and chondrosarcoma^4,5^. The primary endpoint of our study was the prediction of histology- the distinction of chordoma from chondrosarcoma -using a radiomic signature based on MRI, which may be beneficial in preoperative decision-making and reduce unnecessary procedures in selected cases pending prospective validation.

The distinction of real chordoma residual from postoperative/postradiotherapy changes and the high rate of recurrence despite multimodal treatment represent an additional challenge in management. The distinction of real chordoma residual was our secondary endpoint. Current recommendations in the treatment of chordoma are focused on maximal safe surgical resection, and adjuvant high-dose radiotherapy (RT)^6–8^. Gross-total resection (GTR) is usually unachievable due to the infiltration of neurovascular structures. Radiotherapy is the recommended adjuvant treatment after both GTR and incomplete resections of all skull base chordomas^6–8,11^. Patients with local recurrence or metastatic dissemination despite repeated surgery and adjuvant RT might be considered for adjuvant treatment such as targeted therapy and immunotherapy^7–10^.

Regarding prognosis, few studies report prognostic factors for progression-free survival (PFS) and overall survival in skull base chordomas. One of the most significant prognostic factors for increased PFS in chordomas is radical resection^6,7^. Other significant risk factors for poor local chordoma control and decreased survival were identified as: tumor volume^13,14^, location^14,15^, histology (dedifferentiated chordoma)^13,15^, increasing age^13,14^, vascular involvement^15^, and a characteristic molecular/genetic prognostication panel profile (Ki-67,1p36 homozygous deletions, overexpression of CDK4,27 brachyury,15-PDGFR-a, and epigenetic dysregulation)^17,18^. The tertiary endpoint was to develop a robust algorithm capable of predicting 2-year PFS based on an integrated radiomic- clinical model.

In summary, one of the primary objectives of our study was to distinguish chordomas from other clival pathologies, such as chondrosarcomas, using preoperative MRI and radiomic features^19–25^. A secondary objective was to differentiate true chordoma residuals from postoperative changes, and the tertiary endpoint was to predict prognosis as defined by 2-year PFS, using radiomics.

## Results

### Demographic, clinical, and radiological data

Our study was conducted on clinical and radiological data from 61 patients with skull base chordomas (Table 1). The model assessing the chordoma signature was trained against the 100 “skull base background” regions of interest (ROIs) and the cohort of 14 chondrosarcomas. The chordoma and chondrosarcoma patient cohorts did not differ significantly in age, gender, tumor volume, tumor extent, the number of surgeries, or the radicality of resection (Table 1). Among patients with chordoma, 57 patients had a follow-up longer than 2 years and therefore were eligible for the 2-year PFS prognostic study.

**Table 1:** Demographic and clinico-radiological data of chordoma and chondrosarcoma cohorts

	Chordoma	Chondrosarcoma	*p*	
Patients	**61**	**14**		
Age (mean, SD)	50 (SD 14)	53 (SD 18)	*p* = 0.482	^a^
Gender				
Male	33 (54%)	4 (29%)	*p* = 0.087	^b^
Female	28 (46%)	10 (71%)		
Sekhar score without recurrence (mean, SD)	8.8 (SD 3.6)	8.4 (SD 2.8)	*p* = 0.793	^a^
Tumor volume (cm^3^)	27.3 (SD 3.1)	18.2 (SD 2.2)	*p* = 0.445	^a^
Number of surgeries ≥ 2	29 (48%)	5 (36%)	*p* = 0.423	^b^
Radicality/Extent of surgery				
GTR	**20 (33%)**	4 (29%)	*p* = 0.761	^b^
STR/PR	41 (67%)	10 (71%)		
PFS rate at 2 years	37/57 (65%)	10/13 (77%)	*p* = 0.405	^b^
Follow-up (median, range, years)	5 (0.5–25.5)	4.7 (0.4–8.5)	*p* = 0.920	^a^

Among chondrosarcomas, only13/14 had ≥2 years follow-up.

*PFS* progression-free survival at 2 years, *GTR* gross-total resection, *STR/PR* subtotal resection/partial resection, *SD* standard deviation.

^a^*t*-test.

^b^Chi-squared test.

### “Chordoma signature” and prediction of histology from CET1wMRI

Both models, XGBoost and MLP, were trained and tested on a cohort of 73 preoperative MRIs of chordomas, which included 12 artificially generated cases (20%). Similarly, the chondrosarcoma cohort (*n* = 17) included 3 artificially generated cases (20%). Thus, the chordoma signature from a dataset of 73 was assessed against a mixed cohort of 17 chondrosarcomas and 100 skull base background ROIs.

**Fig. 1:**
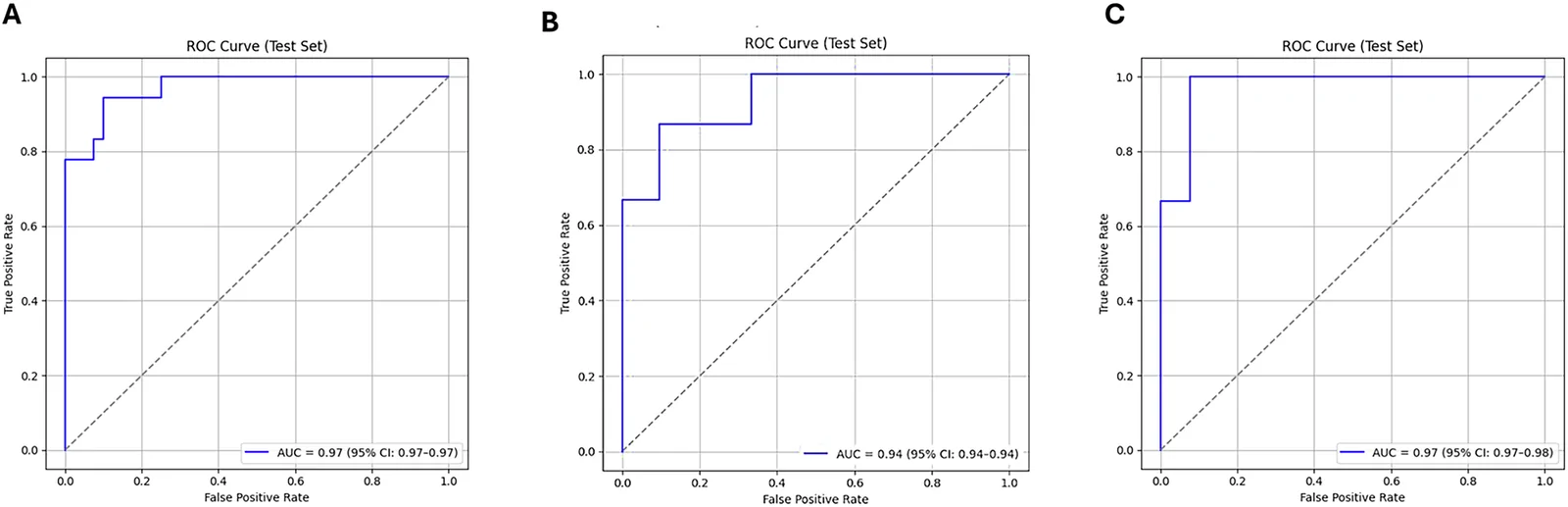
Radiomics XGBoost model accuracy. **A** ROC AUC evaluation of a nested cross-validated XGBoost model for preoperative distinction of chordoma from skull base background and chondrosarcoma. **B** ROC AUC evaluation of the XGBoost model of prediction/distinction of chordoma residual from postoperative changes. **C** ROC AUC evaluation of the XGBoost model for the prediction of 2-year progression-free survival in chordoma. AUC area under the curve, CI confidence interval, ROC receiver operating characteristic.

The nested CV accuracy of the XGBoost model in distinguishing chordoma from skull base background and chondrosarcoma was 0.90 [95% CI: 0.84–0.96], AUC-ROC: 0.97 [95% CI: 0.93–0.97], MSE: 0.08, log loss: 0.16, F1-score: 0.91, precision: 0.95, recall: 0.88 (Fig. 1A). Consistently, comparable performance was observed using a leakage-safe restrictive patient-grouped bootstrapped cross-validation framework, yielding an accuracy of 0.82 [95% CI: 0.77–0.85], AUC-ROC of 0.89 [95% CI: 0.87–0.93], specificity 0.91 [95% CI: 0.87–0.94], and MSE of 0.14 [95% CI:0.11–0.17].

**Fig. 2:**
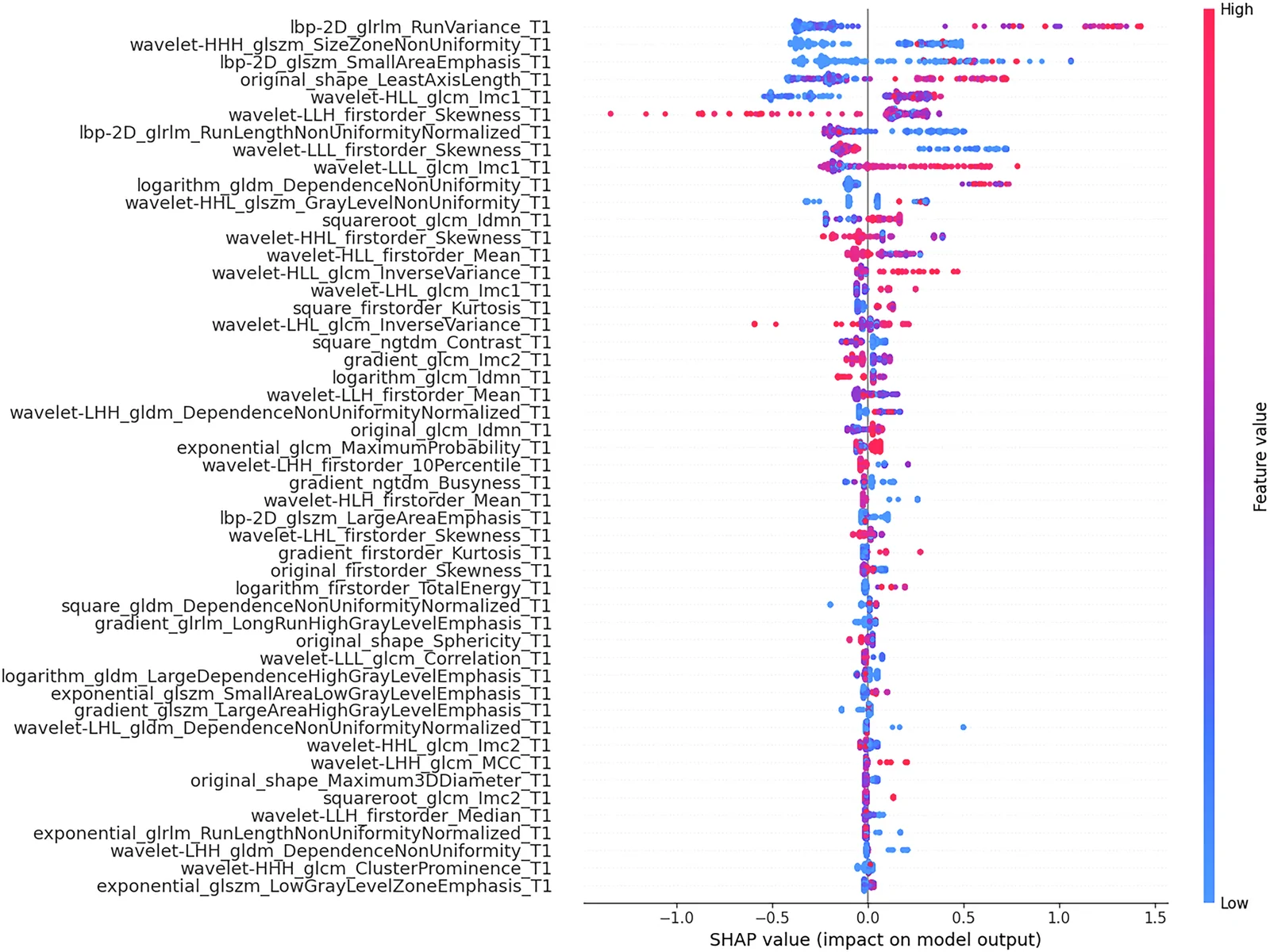
Significance of features in distinguishing between chordoma and chondrosarcoma. Most significant features according to the Shapley values from the XGBoost model of preoperative distinction of chordoma versus chondrosarcoma.

The most challenging part of chordoma signature training was the preoperative distinction of skull base chordoma from radiologically similar chondrosarcoma. A separate XGBoost model for distinguishing between chordoma and chondrosarcoma achieved an accuracy of 0.8 [95% CI: 0.75–0.97], recall: 0.93, precision: 0.89, F1-score: 0.92, log loss: 0.25, MSE: 0.12. AUC-ROC: 0.92 [95% CI: 0.78–0.99]. The most significant features according to the Shapley values from the XGBoost model for preoperative distinction of chordoma versus chondrosarcoma are listed in Fig. 2.

**Fig. 3:**
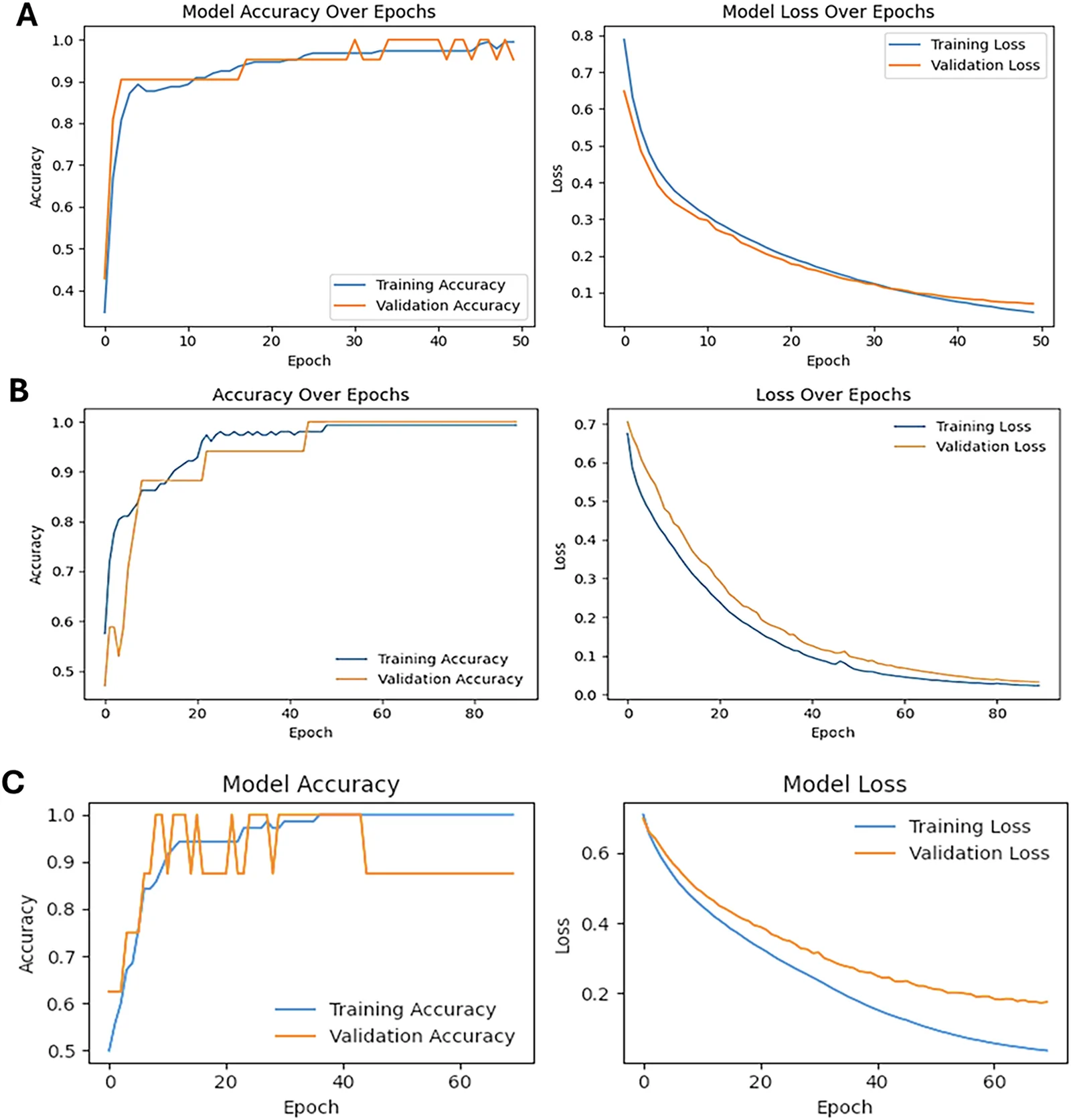
Training and validation performance curves for sequential neural network models. Training and validation accuracy and loss curves from two-way holdout split multilayer perceptron models for (**A**) binary classification of chordoma versus chondrosarcoma, **B** binary distinction of chordoma from postoperative change, and **C** binary prediction of 2-year progression-free survival in skull base chordoma.

Binomial prediction of histology of chordoma versus chondrosarcoma/skull base background using 4-layer MLP with 2-way holdout internal validation achieved weighted accuracy of 0.89 [95% CI: 0.81–0.96], AUC-ROC: 0.85 [95% CI: 0.68–0.99], recall: 0.83, precision: 0.89, F1-score: 0.85 (Fig. 3A). The nested CV MLP model achieved accuracy of 0.8 [95% CI: 0.73–0.85], AUC-ROC: 0.88 [95% CI: 0.72–0.93].

From a histopathological perspective, our cohort included 35 conventional chordomas (57.4%), 14 chondroid chordomas (23%), 1 dedifferentiated chordoma (1.6%), and 11 (18%) unspecified chordomas. Due to the small number of patients (50 patients) with known histology subtype, it was not possible to predict histological subtype on the basis of modeling.

### Radiomics distinction of chordoma residual from postoperative changes

Previously trained XGBoost model describing the chordoma signature was applied to distinguish “unseen” 100 skull base chordoma residuals (ROIs originating from 41 patients with real residuals before radiotherapy) against 80 postoperative changes. The nested CV XGBoost model (after hyperparameter tuning) achieved an accuracy of 0.91 [95% CI: 0.85–0.97], recall: 0.90, F1-score: 0.92, and MSE: 0.1. The ROC analysis showed an AUC of 0.94 [95% CI: 0.93–0.95] (Fig. 1B). Consistently, comparable performance was observed using a leakage-safe restrictive patient-grouped bootstrapped cross-validation framework, yielding an accuracy of 0.89 [95% CI: 0.84–0.93], AUC-ROC of 0.95 [95% CI: 0.89–0.98], specificity of 0.97 [95% CI: 0.93–0.99], and MSE of 0.09 [95% CI: 0.0.6–0.13].

In comparison, a 4-layer MLP with 2-holdout with internal validation for binary classification (chordoma residual vs. postoperative changes) attained a weighted accuracy of 0.90 [95% CI: 0.80–0.97], AUC 0.92 [95% CI: 0.81–0.99], recall: 0.75, F1-score: 0.86, and MSE: 0.09 (Fig. 3B). The nested CV MLP model achieved accuracy of 0.75 [95% CI: 0.68–0.79], AUC-ROC 0.8 [95% CI: 0.72–0.88] in final test-set.

### Prediction of prognosis using an integrated radiomic-clinical model

Both models, XGBoost and MLP models, were trained and tested to predict the 2-year PFS of chordomas. Overall, 57 out of 61 patients with skull base chordomas had follow-up longer than 2 years and were eligible for the prognosis study. The models were trained with an additional 17 (30%) artificially generated chordoma cases, which narrowed the confidence intervals of the XGBoost model by 8%.

The accuracy of the XGBoost model with a two-way holdout split was 0.95 [95% CI: 0.94–0.95]. The nested CV XGBoost model, after hyperparameter tuning, achieved an accuracy of prediction of 0.87 [95% CI: 0.74–0.98], precision: 0.85, recall: 1.0, F1-score: 0.92 [95% CI: 0.91–0.93], and MSE: 0.07. The AUC from ROC analysis was 0.97 [95% CI: 0.92–0.98] (Fig. 1C).

The four-layer MLP model using a two-way holdout split achieved a weighted accuracy of 0.93, loss of 0.17 after 90 iterations, recall: 0.9, and F1-score: 0.9 (Fig. 3C). The nested 3/5-fold CV MLP achieved an accuracy of 0.75 [95% CI: 0.62–0.88] and an AUC of 0.76 [95% CI: 0.54–0.97].

**Fig. 4:**
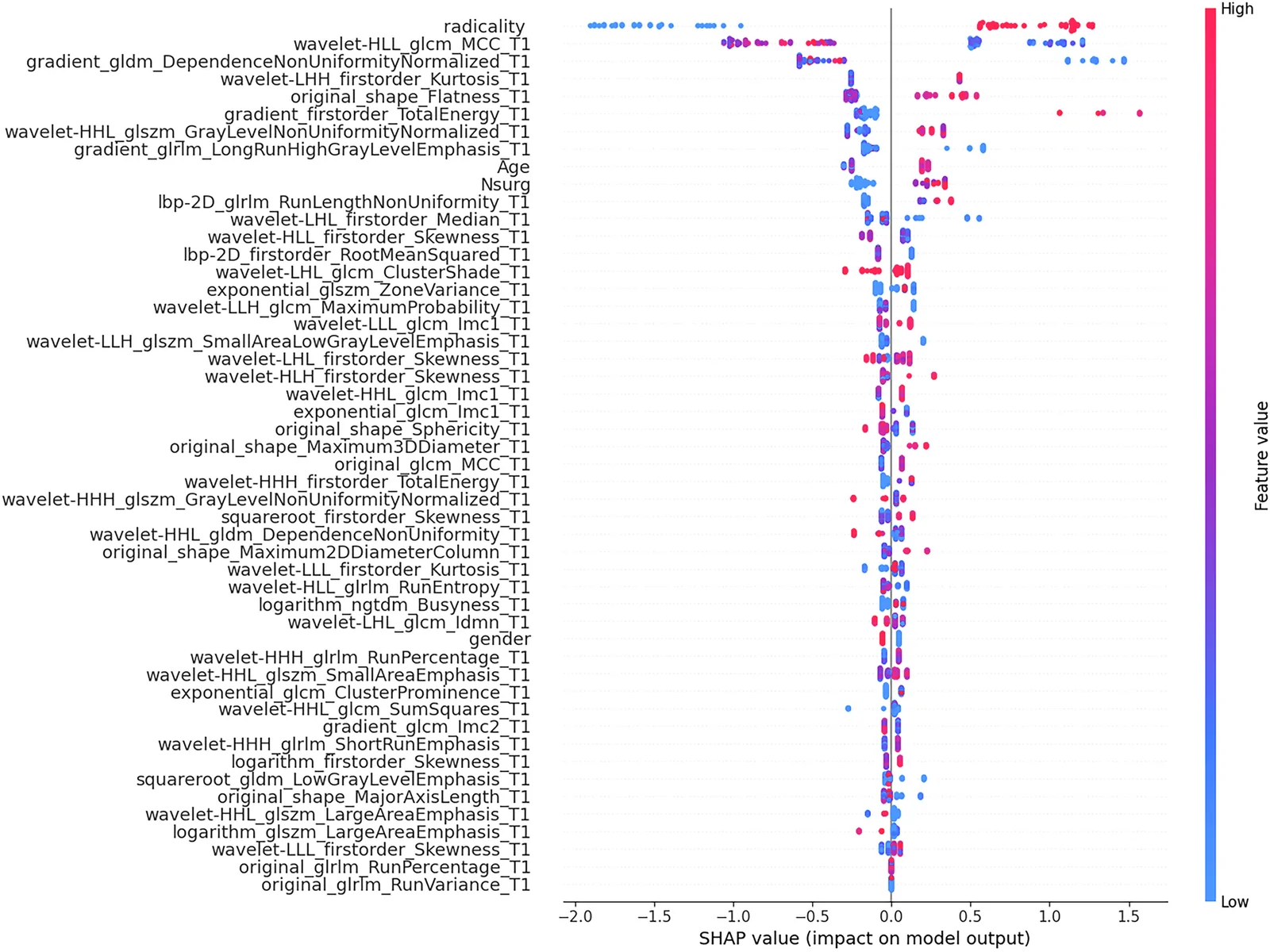
Significance of features in predicting prognosis. Most significant features, according to the Shapley values from the XGBoost model of prediction of prognosis based on the integrated radiomic-clinical model.

Demographic (age, gender) and clinical variables (histology, number of surgeries, radicality of last surgery, type of radiotherapy) considered to be prognostic factors for recurrence were integrated with the radiomic features, creating a “multimodal” matrix. In addition to texture features (predominantly wavelet-based), incomplete resections, re-operations, older age, male gender, tumor size (max2D- and max3D_Diameter), and irregular shape (defined as lower sphericity, higher flatness features) were detected as significant contributors to the final prediction model for growth (based on Shapley values) (Fig. 4).

## Discussion

Radiomics and machine learning are increasingly integrated into clinical workflow across various oncological pathologies^26–30^. In the context of skull base tumors, radiologic differentiation between chordomas and chondrosarcomas remains a significant challenge due to their overlapping radiological features and somatotopy. Despite having distinct biological behaviors and prognoses, they often appear indistinguishable on standard MRI.

Our models demonstrate that radiomics, through high-dimensional feature extraction and advanced classification models, can detect a specific chordoma signature and achieve high diagnostic accuracy for multiple clinically relevant endpoints: histological classification and distinction of residual tumor from postoperative changes. While Buizza et al. reported application of radiomics and clinical features in prediction of local chordoma control after radiotherapy^31,32^, our work is the first that attempts to define the radiomic signature of chordoma to predict the existence of residual tumor following surgery and radiotherapy and to determine prognosis.

Our algorithms could be integrated as a support tool across all stages of chordoma management: e.g., as a radiological assistant in distinguishing true residual tumor from postoperative changes, or as a surgical decision-making assistant to help reduce preoperative uncertainty or unnecessary re-operation, when facing the dilemma of residual disease versus postoperative/postradiotherapy changes. Moreover, the algorithm involved in the prediction of PFS based on preoperative MRI could triage high-risk chordomas and support a patient-tailored surveillance protocol.

Interestingly, our model predicting the natural course of 2-year PFS identified tumor size and shape-related features (sphericity, flatness) as significant predictors. Moreover, a few of the known clinical predictive factors, such as incomplete resection, re-operations, older age, and male gender, were found to be significant contributors to the model. In the future, we aim to build models capable of detecting molecular therapeutic targets (e.g., methylation, tyrosine kinase receptors).

One major limitation of our study was the small cohort size, reflecting the rarity of chordomas. Small datasets increase the risk of overfitting, reduced generalizability, and a lack of robustness on unseen cases, especially when the validation set is limited. To mitigate this, we included data from two centers and augmented the dataset by 20% using standard geometric transformations, which improved model accuracy by 4–6% without signs of overfitting. In contrast, using only the original dataset or a 10% augmented version led to overfitting. Similarly, the prognostic analysis, based on just 57 cases, required 30% augmentation to reduce overfitting. It is recommended that up to 20–30% simulated data can be utilized in order to rationalize the training process, while limiting bias^21,22^. Continued data collection is needed to strengthen the model.

Conversely, including MRI data from three scanner types across centers increased heterogeneity and helped improve the generalizability of our model. We show that a model based on a small heterogeneous cohort can achieve reliable performance, based on standard MRI sequences. Future collaborations with additional tertiary centers are planned to further expand the dataset and increase protocol diversity.

To overcome the limitations of traditional convolutional neural networks, including their requirement for large datasets and limited interpretability, we used a hybrid approach combining radiomics-based features with a lightweight supervised neural network (MLP). This allowed us to retain both interpretability and performance. Notably, key features were consistently selected across models, supporting their relevance. Our radiomics model achieved diagnostic accuracy superior to 0.9 in distinguishing chordomas from chondrosarcomas, and chordoma residuals from postoperative changes. Our models outperformed the previously published work, which achieved significantly lower accuracy (0.6 with 95% CI: 0.5–0.8)^31^.

We further addressed the issue of a small dataset by integrating nested CV into the machine learning XGBoost algorithm. This approach is computationally more demanding, but decreases the risk of overfitting arising from using the same dataset for both tuning and performance measurement^24^. We found similar good performance using different *k* = 4–7 outer folds and 4–5 inner folds. Nested CV reduced optimistic bias in performance estimation; we additionally used bootstrapping/repeated CV for confidence intervals. Additionally, we minimized overfitting by using patient-level grouped nested cross-validation, ensuring that all ROIs and augmented samples from each patient were confined to a single fold. Performance robustness was further supported by within-fold preprocessing and a patient-level permutation test confirming discrimination beyond chance.

Another limitation includes the disproportion between the number of patients and the studied features. Although the conventional 10:1 “events per variable” rule was relaxed, machine learning techniques like XGBoost inherently handle high-dimensional feature spaces through embedded feature selection during tree construction and regularization (L1/L2), without requiring separate pre-training feature selection^33,34^. Our model used standard XGBoost tuning parameters, including L1 and L2 penalization, maximum tree depth, minimum sum of weights, learning rate, gamma/minimum split loss, and class weight scaling^23,24^. The definition of the tuning parameters is beyond the scope of this publication.

A third limitation concerns the definition of ground truth for residual tumors versus postoperative changes. In the absence of histologic confirmation, this distinction was based on an expert radiologist’s review. Histological confirmation was available in only 28 patients (46%). In our cohort, 13 patients with postoperative residuals (21%) and 20 patients with postoperative changes (33%) did not have histological confirmation of “ground truth”. For these 33 patients, ground truth was based on longitudinal radiologic surveillance (e.g., lesion growth or stability) and comparison of radiological appearance/residual location with preoperative scans. This may introduce diagnostic-review (incorporation) bias, as readers were not fully blinded to clinical context or prior imaging, potentially leading to optimistic performance estimates of specificity by reducing apparent false-positive classifications. To mitigate this limitation, non-histology cases were adjudicated using predefined imaging criteria (e.g., unchanged location and appearance, volumetry) and supported by serial surveillance. External validation in independent cohorts with blinded assessment and greater histologic verification remains essential to confirm generalizability.

Similarly, the semi-automated segmentation of chordoma ROIs performed by neurosurgeons may represent a possible source of bias, especially in the presence of irregular shape and heterogeneous contrast enhancement. However, our high inter-rater DICE scores suggested consistency and high reliability.

The final limitation was insufficient compliance with surveillance, affecting the prediction model for prognosis. Surveillance imaging was available for 57 patients (93%) at 2-year follow-up (FU) and only 31 patients (51%) at 5-year FU. This significant change is multifactorial: a higher proportion of chordomas were operated on recently, and non-compliance increases with the duration of scheduled FU. In addition, loss of FU due to mortality (both related and unrelated to chordoma) was 16% (10 patients), with a median time of death of 2.5 years [0.3–25.5 years].

Our MRI-based machine learning model demonstrates high performance in distinguishing chordoma from chondrosarcoma, differentiating postoperative residual tumor from treatment-related changes, and predicting prognosis. A potential future application of this chordoma signature may be as a clinical decision-support tool to reduce diagnostic uncertainty and assist multidisciplinary planning, particularly in cases where invasive diagnostic procedures or repeat interventions are being considered. Furthermore, automated detection and segmentation of chordoma could support radiologic assessment during long-term postoperative surveillance. Future multi-institutional validation will be essential to improve robustness and facilitate clinical translation.

## Methods

### Demographic data, study design

**Fig. 5:**
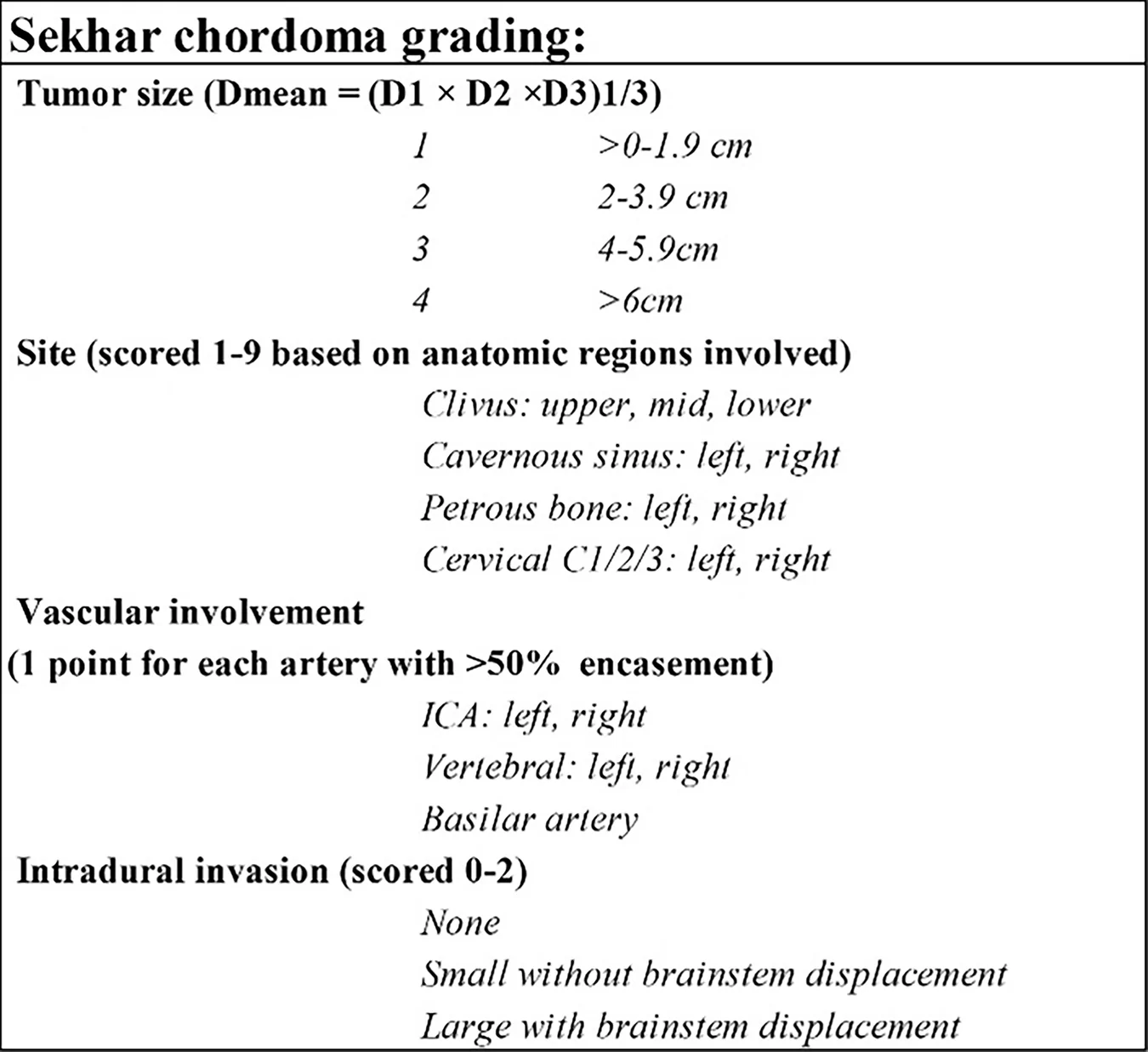
Modified Sekhar’s score. The recurrence sub-score was excluded. Internal carotid artery (ICA).

This retrospective study included 61 patients with primary skull base chordoma and a comparator cohort of 14 skull base chondrosarcomas treated surgically at two tertiary centers between January 1998 and December 2023. Clinical, radiological, perioperative, and histopathological data were collected (Table 1). Radiological variables included the extent of chordoma based on the modified chordoma grading score proposed by Sekhar et al.^15^ and radiomic features (Fig. 5). The Sekhar grading system was modified by excluding the recurrence sub-score, such that the score reflected tumor size and anatomical extent rather than clinical behavior. This study was performed in accordance with the Declaration of Helsinki and its subsequent amendments. Institutional Review Board (IRB) approval was obtained at both participating institutions: IRB Mass General Brigham, #60768218; ERIN for Cambridge University Hospitals NHS Foundation Trust, R&D#: A097411. Written informed consent was waived due to the retrospective nature of the study.

The primary endpoint was to identify a radiomic signature specific to chordoma and evaluate its accuracy in predicting histology from preoperative MRI. In order to improve our chordoma signature detection, we trained the chordoma model not only on skull base background, but also on a comparable cohort of skull base chondrosarcomas, which are a distinct pathology sharing similar anatomic affinity, growth pattern, and radiologic profile with chordoma. The demographic and clinical characteristics of chondrosarcomas are included in Table 1.

Using this radiomic chordoma signature, the secondary endpoint assessed the model’s ability to automatically distinguish chordoma residuals from postoperative/postradiotherapy changes. The tertiary endpoint focused on the accuracy of predicting 2-year PFS based on radiomics.

### Radiomics

Our radiomics algorithm was written in Python (version 3.12, Python Software Foundation, Keras).

#### Image acquisition and pre-processing

Anonymized and masked pre- and postoperative 3D contrast-enhanced T1-weighted magnetic resonance images (CET1wMRI) were collected for each patient. The images were acquired on different MRI systems with a field strength of 1.5 T (Philips, GE, Siemens). The axial 3D-CET1wMRI sequence was acquired with the following parameters: TR/TE 465–800/9–15 ms, number of excitations (NEX) = 2, matrix size 320 × 320 (reconstructed to 512 × 512), voxel spacing 0.35 × 0.35 × 1–2.5 mm, flip angle 10–20°, and acquisition time 3–4.5 min. Thirteen patients (21%) with different voxel spacing and dimensions of CET1wMRI images were rescaled to a 0.3516 × 0.3516 × 1 matrix using B-spline interpolation. Subsequently, we performed bias field correction using the N4 method, z-score intensity normalization, and image filtering.

#### Cohort design

The chordoma signature model was based on 61 segmented chordoma cases and was trained against chondrosarcomas and skull base background areas. Due to the small size of the cohort (*n* = 61), the data were augmented with an additional 20% artificially generated cases (*n* = 12) using restricted geometric transformations of the original MRI, such as cropping (<20%), in-plane flips, rotations (±5–20°), zooming (±10–20%), undulation/elastic deformation (*α* ≈ 5–10 voxels), and translations (≤5–10 voxels)^21,22^. Similarly, the cohort of 14 chondrosarcomas was augmented by 20%, adding 3 artificially generated cases. The skull base background areas were created by segmenting random regions of the skull base on the CET1wMRI of chordoma patients, with volumes comparable to those of the tumors.

In the model distinguishing the real chordoma residual from postoperative changes, 100 skull base chordoma residual ROIs were segmented from postoperative 3D-CET1wMRI of 41 patients with incomplete resection. These data were trained against 80 segmented areas of confirmed postoperative changes, identified by radiologists. In the prognosis study (*n* = 57), an additional 30% artificial chordoma cases were generated (*n* = 17), as 10 and 20% of augmentation were found to be insufficient to avoid model overfitting.

#### Feature extraction

Features were extracted from the ROI, which included only voxels containing chordoma. The ROI was created using semi-automated segmentation with an active contour algorithm (ITK-SNAP) by skull base neurosurgeons (D.S., R.M., R.C.). The Dice similarity coefficients (DSC) compared the rate of overlap in segmentations between different assessors (D.S. vs R.M. for the UK dataset, D.S. vs R.C. for the US dataset). The DSC were 0.94 and 0.88, respectively, indicating good inter-rater agreement.

The distinction between true chordoma residual and postoperative changes was more challenging. The ground truth was based primarily on histopathological and radiological reports. Among 41 patients who underwent incomplete resection, direct histopathological confirmation of true residual tumor was obtained in 28 patients: via re-operation in 26 patients (63%), and by biopsy in an additional 2 patients(5%). In the remaining 13 patients (32%), the presence of residual was estimated by expert radiological opinion based on its unchanged appearance and location in comparison with preoperative MRI. Additionally, the remaining 20 patients from the 61-chordoma cohort had complete resection, with only postoperative/postradiotherapy changes according to the expert radiology and oncology panel reviews.

Features were constructed as the subsequent combination of three processes: initial image filtering (3D single-decomposition Haar wavelet transform, Laplacian of Gaussian, square, square root, logarithm, exponential, and local binary pattern 2D), followed by the extraction of texture or directional intensity-based features, superposed with the extraction of morphological or histogram-based features.

The texture features consisted of 11 gray-level co-occurrence matrix (GLCM) features, 13 gray-level run-length matrix features, 13 gray-level-size-zone-matrix features, 16 neighboring gray-level dependence matrix features, and 5 neighborhood gray-tone difference matrix features. In total, 1410 combined features were extracted per ROI from the CET1wMRI sequence^19–22^. The name of each feature encodes the process of calculation, for instance, CET_HHL_GLCM_Kurtosis represents the kurtosis feature extracted from a 3D HHL Haar wavelet-decomposition of a CET1wMRI.

#### Prediction models

Several models were evaluated in the process, and we report two best-performing prediction models : (1) Classic radiomics XGBoost model and (2) four-layer standard feedforward Multilayer Perceptron (MLP)^23–25,35^. The XGBoost model (eXtreme Gradient Boosting) is a machine learning algorithm implementing gradient-boosted decision trees^23,24^. During the training and tuning of the XGBoost model, we applied various combinations of outer/inner folds in a nested cross-validation (CV) method. The best results were achieved with 5-outer/5-inner folds. In detail, the nested CV involved repeatedly and randomly splitting the dataset into training:testing sets, referred to as the “outer folds” (4:1 split). Within each outer training set, the data were further divided into “inner folds**”** (training-to-validation ratio) for model building and hyperparameter tuning. Finally, the performance of the model (including confidence intervals) was evaluated on an “unseen” testing dataset, supporting robustness and generalizability^24^. Confidence intervals of the test dataset were assessed using bootstrapping (1000 iterations). To address the imbalance between feature dimensionality and cohort size, XGBoost models were trained with strong regularization and constrained complexity. Key hyperparameters (*max_depth*, *min_child_weight*, *subsample*, *colsample_bytree*, *reg_alpha/reg_lambda*, and *scale_pos_weight*) were tuned within the inner loop of nested cross-validation to reduce optimistic bias and overfitting.

To prevent data leakage and overly optimistic performance estimates, CV was also performed at the patient level rather than at the ROI level (patient-grouped nested CV strategy). All ROIs and augmented variants from the same patient were confined to a single outer fold within a patient-grouped nested CV framework, ensuring complete separation between training and testing data. Accordingly, model evaluation reflects true generalization to unseen patients rather than repeated sampling of correlated ROIs, which is particularly important in small datasets with synthetic augmentation. Patient-grouped cross-validation was treated as the primary evaluation, as it best reflects real-world deployment on independent patients in comparison with the more optimistic nested CV.

Evaluation of the learning XGBoost model was also based on the average area under the curve (AUC) from receiver operating characteristic (ROC) analysis, the mean squared-error (MSE), F1-score, and confusion matrix (accuracy, recall/sensitivity, specificity). Feature significance was assessed using Shapley scores and the F-score from the XGBoost model. Model interpretability was further assessed using SHapley Additive exPlanations (SHAP), with mean absolute SHAP values used to rank the most influential radiomic features driving chordoma discrimination.

The MLP was a feedforward neural network composed of 3 fully connected hidden layers (32–64–32-neurons), followed by a single output neuron, integrating the Adam optimizer and ReLU activation function^25,35^. Training was conducted over 90 epochs with early stopping, learning_rate of 0.001, and a batch size of 8. Two different setups were applied during training of the MLP model: (1) simple 2-way holdout split with internal validation (train:validation:test split 81%:9%:10%), and (2) nested 3/5-fold cross-validation was implemented. Nested CV is a more robust cross-validation compared to 2-way holdout; however, it is more sensitive to limited data. The performance of the MLP model was assessed from training and validation curves of both loss and accuracy, along with the AUC-ROC method, weighted accuracy, recall, F1-score, and precision. The 32–64–32 architecture was used as a compact, highly L2 regularized MLP and was selected empirically during preliminary experiments as the best-performing low-capacity configuration under early stopping.

Finally, the XGBoost algorithm was used to train the integrated radiomic-clinical model of prognostic factors for PFS, which was based on simultaneous analysis of radiomic features along with demographic (age, gender) and clinical variables (histology, number of surgeries, radicality of the last surgery, type of radiotherapy).

#### Statistics

Univariate analysis included the *chi-*square test and *t-*test, assessing the difference between the chordoma and chondrosarcoma datasets. All measurements were assessed at a 95% confidence level; *p* < 0.05 was considered statistically significant.

## Data Availability

The data that support the findings of this study are available from the University of Cambridge, but restrictions apply to the availability of these data, and so they are not publicly available. Data are, however, available from the authors upon reasonable request and with permission of the University of Cambridge.

## Code availability

The underlying code for this study [and training/validation datasets] is not publicly available but may be made available to qualified researchers on reasonable request from the corresponding author.
